# Depression and compliance in hemodialysis patients: pilot study

**DOI:** 10.1192/j.eurpsy.2025.1239

**Published:** 2025-08-26

**Authors:** S. Mizera, A. Osuch, A. Bąk, T. Cyganek, M. Zatorski, D. Gojowy, M. Adamczak, K. Krysta, A. Więcek

**Affiliations:** 1 Doctoral School; 2 Medical Students’ Association, Department of Nephrology, Transplantation and Internal Medicine, Medical University of Silesia; 3 SWPS University of Social Sciences and Humanities; 4 Department of Nephrology, Transplantation and Internal Medicine; 5 Department of Rehabilitation Psychiatry, Medical University of Silesia, Katowice, Poland

## Abstract

**Introduction:**

Lack of compliance is well-known limiting factor in achievement of the therapeutic targets in medical care. The frequency of noncompliance as well as the factors contributing to this condition are currently not well understood. In hemodialyzed patients lack of precise adherence to medical recommendations is particularly important for long time survival. Depression occurrence may have an adverse impact on the medical compliance of these patients.

**Objectives:**

The aim of this study was to analyze prevalence of depression symptoms and its impact on compliance to medical recommendations in patients on chronic hemodialysis.

**Methods:**

Forty (M=26;F=14) patients undergoing routine hemodialysis session have taken part in a two-part survey consisting: Beck’s Depression Inventory and IMB-Q - The Information-Motivation-Behavioural Skills Questionnaire. IMB test includes three subcategories as a basis for tailoring of the model to an individual health-related behavior – a) information about disease and treatment, b) motivation and c) behavioral skills to comply with treatment. Additionally, in all patients weight gain between dialysis session was analyzed.

**Results:**

Seventeen patients (47.5%) fulfilled depression criteria, out of which 5 (12.5%) had moderate or severe grade disease. Depression symptoms were inversely correlated with behavioral skills to comply with treatment measured by IMB-Q test in men (r= -0,416; p=0,034). There was no significant correlation between results of Beck Depression Inventory and body mass change between dialysis sessions. There was also no significant difference in the interdialytic weight gain between patients with and without depression.

**Conclusions:**

Depression is frequently found in male patients with end stage kidney disease treated with chronic hemodialysis. Male hemodialysis patients with depression are less likely to be compliant to medical recommendations because of weaker behavioral skills. Improvement of the compliance in hemodialysis patients may play an important role in the prolongation of life in these group.

**Disclosure of Interest:**

None Declared

